# Hypoalbuminemia and cisplatin-induced acute kidney injury

**DOI:** 10.3389/fphar.2024.1510477

**Published:** 2024-12-11

**Authors:** Wen-Jun Yin, Yu-Jie Huang, Qing Zhu, Xiao-Qing Lin, Hui-Ling Piao, Qian-Qian Yu, Chang-Hong Lai, Guang-Liang Zhou, Ling-Yun Zhou, Kun Liu, Xiao-Cong Zuo, Shan-Ru Zuo

**Affiliations:** ^1^ Department of Pharmacy, The Third Xiangya Hospital, Central South University, Changsha, Hunan, China; ^2^ Department of Pharmacy, Hunan Cancer Hospital & The Affiliated Cancer Hospital of Xiangya School of Medicine, Central South University, Changsha, China; ^3^ Center of Clinical Pharmacology, The Third Xiangya Hospital, Central South University, Changsha, Hunan, China

**Keywords:** cisplatin, acute kidney injury, hypoalbuminemia, meta-analysis, retrospective study

## Abstract

**Background:**

Cisplatin binds to serum albumin in the body at a rate of 90%, and high levels of free cisplatin are a significant cause of its nephrotoxicity. Therefore, hypoalbuminemia theoretically poses a significant risk factor for cisplatin-induced acute kidney injury (CIA) and can be easily corrected. However, existing research results are inconsistent. Our aim is to confirm the association between hypoalbuminemia and CIA through a meta-analysis and a dual-center real-world data study.

**Methods:**

First, we used a random-effects meta-analysis to summarize the odds ratio (OR) of the risk relationship between hypoalbuminemia and CIA. Then, we conducted a retrospective analysis of patients using cisplatin from Xiangya Third Hospital of Central South University (2014–2023) and Hunan Cancer Hospital (2019–2023) to analyze the relationship between hypoalbuminemia and CIA.

**Results:**

The meta-analysis, which included six studies involving 4,359 cases, showed that hypoalbuminemia is associated with an increased risk of CIA (OR, 2.13; 95% CI, 1.37–3.32). A total of 5,452 and 25,214 patients from Xiangya Third Hospital and Hunan Cancer Hospital, respectively, were included. Both centers found a significant association between hypoalbuminemia and an increased risk of CIA (OR, 2.76; 95% CI, 1.94–3.93; OR, 2.88; 95% CI, 2.17–3.81), and the sensitivity analysis results were consistent.

**Conclusion:**

Through meta-analysis and dual-center real-world data studies, we confirmed that hypoalbuminemia is an independent risk factor for CIA. Therefore, it is recommended that patients using cisplatin undergo serum albumin level testing and regular monitoring during treatment. Actively adjusting albumin levels may reduce the risk of CIA.

## 1 Introduction

Cisplatin is a classic platinum-based first-line antitumor drug (Romani, 2022). However, its clinical application is limited due to its severe adverse effects (Hussain et al., 2012; Ismaili et al., 2011; Moxley and McMeekin, 2010; Lee et al., 2007; Wagner et al., 2016). Cisplatin-induced acute kidney injury (CIA) is a common and severe adverse effect of cisplatin, with an incidence rate of 10%. Studies have shown that 45% of CIA patients develop chronic kidney disease (Faig et al., 2018), and CIA patients have longer hospital stays and a 2.71-fold increased risk of mortality (Hod et al., 2021). As there are no definitive and effective treatments for CIA, identifying high-risk populations and early intervention are crucial to reducing its incidence.

High free cisplatin concentration is an important factor in its nephrotoxicity. High free cisplatin concentrations increase the exposure time and damage to renal tubular cells, leading to acute kidney injury (Meng et al., 2017; Holditch et al., 2019). In the initial phase I and II clinical studies of cisplatin, more than 50% of patients experienced nephrotoxicity (Mandic et al., 2003; Rybak et al., 2009). Although the incidence of CIA has significantly decreased with hydration interventions in recent years, the rate remains high (Lee et al., 2007; dos Santos et al., 2012; Hoek et al., 2016). Hydration dilutes free cisplatin in the plasma and increases urine output, promoting the excretion of free cisplatin and ultimately reducing its damage to renal tubular cells.

Hypoalbuminemia is an established independent predictor of AKI and AKI-related mortality in various clinical settings, with potential mechanisms involving systemic inflammation and reduced renal perfusion (Wiedermann et al., 2010). However, drug-induced AKI, such as CIA, and general AKI have distinct pathophysiological mechanisms and risk factors. For example, the mechanisms of CIA are mainly cisplatin direct nephrotoxicity, DNA damage, and oxidative stress (Tang et al., 2023), sepsis AKI is predominantly an infection and systemic inflammatory response (Umbro et al., 2016). While hypoalbuminemia is a well-known risk factor for AKI in critical illness, its role in CIA remains unclear.

Ninety percent of cisplatin binds to serum albumin in the body. Bound cisplatin exists in a more stable form in the blood, reducing its toxicity in the free state. The binding of cisplatin allows serum albumin to transport it more effectively throughout the body, reducing its local concentration in the kidneys and thereby mitigating nephrotoxicity (Chen et al., 2018; Chen et al., 2023; Chen et al., 2016). Therefore, theoretically, if patients have hypoalbuminemia, the concentration of free cisplatin will increase, and the risk of kidney damage will also rise.

Although 90% of cisplatin binds to serum albumin, theoretically reducing its free concentration and nephrotoxicity, the specific relationship between hypoalbuminemia and CIA has not been comprehensively evaluated. In two large real-world data-based models for predicting cisplatin-induced kidney injury, hypoalbuminemia was reported as an important predictive variable (Motwani et al., 2018; Okawa et al., 2022). However, some studies have found that hypoalbuminemia is not a risk factor for CIA (Yoshida et al., 2014). Current literature on hypoalbuminemia and CIA comprises observational studies, mostly single-center small-sample retrospective studies. Due to ethical issues, cohort studies are challenging to conduct, and the association between hypoalbuminemia and CIA remains unclear.

Given that hypoalbuminemia is a modifiable risk factor, understanding its precise role in CIA could have significant clinical implications. If hypoalbuminemia is confirmed as an independent risk factor, therapeutic interventions such as human serum albumin infusion could be employed to reduce the incidence and severity of CIA. This study aims to bridge the current knowledge gap by evaluating the association between hypoalbuminemia and CIA through a dual-center large-sample real-world data study and meta-analysis. The findings of this research could contribute to early identification of high-risk patients, more targeted preventive measures, and timely clinical interventions, ultimately improving patient outcomes.

## 2 Methods

### 2.1 Meta-analysis

#### 2.1.1 Eligibility criteria

We included studies that investigated the association between hypoalbuminemia and CIA. Given the susceptibility of observational studies to confounding variables, only those clinical investigations that assessed the impact of serum albumin through multivariate approaches were incorporated into the meta-analysis. Studies were excluded if it was not possible to extract OR and their CI, or if any relevant association metrics could not be recalculated based on the raw data presented in the study reports.

#### 2.1.2 Literature search and study selection

Two authors independently searched PubMed, EMBASE, and Web of Science for studies published in English up to January 10, 2024, relevant to the association between hypoalbuminemia and CIA. The search strategy is detailed in Supplementary Table S1. Initially, they excluded studies unrelated to hypoalbuminemia or CIA based on titles and abstracts. Subsequently, full texts of potentially relevant articles were retrieved and assessed. Hypoalbuminemia was defined as a serum albumin level below 35 g/L. The methodological quality of selected studies was evaluated and scored using the AHRQ bias assessment tool, and the quality of the included studies was assessed using the Newcastle-Ottawa Scale (NOS).

#### 2.1.3 Data extraction and statistical analysis

For each included study, we extracted the following information: (i) the first author’s name, year of publication, country, and period of study; (ii) the definition of hypoalbuminemia; (iii) the total number of participants and the data source; (iv) the association between hypoalbuminemia and CIA, as indicated by the reported multivariable OR and CI. Data extraction was independently conducted by two authors, with any discrepancies resolved through discussion among all researchers involved in the analysis.

Given the anticipated heterogeneity stemming from variations in CIA definitions, cisplatin dosages, and characteristics of study populations, we employed a random-effects analysis to generate pooled effect estimates from the eligible studies. This approach allowed for the consideration of both inter-study and intra-study variability. The pooled ORs and their corresponding 95% CIs were calculated to quantify the association between hypoalbuminemia and CIA. A significant heterogeneity was indicated by an I^2^ statistic exceeding 50%. Forest plots were utilized to graphically represent the results of the meta-analysis.

### 2.2 Real-world data study

#### 2.2.1 Study design

This dual-center retrospective study was conducted on patients treated with cisplatin at the Third Xiangya Hospital of Central South University from January 2014 to December 2023 and the Hunan Cancer Hospital from January 2019 to October 2023. The Third Xiangya Hospital is a tertiary general hospital, while the Hunan Cancer Hospital is a tertiary cancer specialty hospital. The Ethics Committee of the Third Xiangya Hospital approved this study (Approval No. 23829).

Inclusion criteria were as follows: cancer patients aged ≥18 years who received cisplatin treatment and had serum creatinine levels measured within 2 weeks before and after cisplatin administration. Exclusion criteria included patients who developed tumor lysis syndrome (TLS), underwent dialysis, had missing data exceeding 30%, or lacked any serum creatinine measurement before or after cisplatin treatment. Patient recruitment was facilitated through an electronic medical record search at all participating institutions, with data collected according to a pre-established data collection plan.

#### 2.2.2 Data collection

Patient data encompassing gender, height, weight, age, serum levels of uric acid, urea, albumin, calcium, pre- and post-treatment serum creatinine values, as well as diagnostic information, were systematically collected.

CIA was defined according to the nephrotoxicity criteria established by the National Cancer Institute, which identifies CIA as an increase in serum creatinine greater than 0.3 mg/dL or more than 50% above baseline values within 2 weeks following cisplatin administration. Hypoalbuminemia was defined as serum albumin levels below 35 g/L. Based on the severity of albumin depletion, hypoalbuminemia was classified into mild and severe categories: mild hypoalbuminemia was indicated by albumin levels between 25–35 g/L, severe hypoalbuminemia by levels below 25 g/L. The elderly population was defined as individuals aged 60 years and above.

#### 2.2.3 Statistical analysis

Continuous variables are presented as mean ± standard deviation, while categorical variables are summarized as percentages. Differences between categorical variables were assessed using the Chi-square test, whereas the Student's t-test was employed for continuous variables, with a statistical significance threshold set at a two-sided *P*-value of <0.05. For handling missing data, multiple imputation was conducted using the “Predictive Mean Matching” package in R. The association between hypoalbuminemia and CIA was analyzed using logistic regression. To account for multicollinearity and informed by clinical experience, three logistic regression models were constructed: Model 1 was unadjusted; Model 2 adjusted for age, body mass index (BMI), and gender; and Model 3 further adjusted for pre-medication creatinine levels, uric acid, and urea. Additionally, albumin levels were categorized for subgroup analyses. All data analyses were performed using R version 4.2.2. Full access to the research data was granted to all authors, who reviewed and approved the final manuscript.

## 3 Results

### 3.1 Meta-analysis

In total, 712 potentially relevant articles were identified: 138 from PubMed, 364 from Embase, and 210 from Web of Science. Ultimately, six studies involving 4,359 participants were selected for inclusion in the meta-analysis after evaluation of 10 full-text articles (Supplementary Figure S1). The characteristics of the included studies are summarized in Table 1. Given the observational nature of these studies, the quality of the included studies was assessed using the Newcastle-Ottawa Scale (NOS), with the evidence quality concerning the association between hypoalbuminemia and CIA being deemed moderate (Supplementary Table S2). The Agency for Healthcare Research and Quality (AHRQ) bias risk summary is presented in Supplementary Figure S2, indicating that most studies had a moderate risk of bias, with only one study classified as low risk.

**TABLE 1:** Summary of included studies.

Author	Year	Hypoalbuminemia, g/L	Patients	AKI	Study duration	Location	Database/source
Yamamoto et al. (2017)	2017	<35	112	30	2009–2015	Japan	Aichi Medical University School of Medicine
Motwani et al. (2018)	2018	<35	2,118	288	2000–2016	America	Massachusetts General Hospital
Okawa et al. (2022)	2022	<35	1,014	184	2006–2013	Japan	Fujita Health University Hospital
de Jongh et al. (2003)	2003	<35	400	29	1990–2001	Netherlands	Erasmus University Medical Center Rotterdam
Kobayashi et al. (2016)	2016	<35	219	30	2011–2014	Japan	Hokkaido University Hospital
Yoshida et al. (2014)	2014	<35	496	73	2009–2011	Japan	National Cancer Center Hospital East

**FIGURE 1:**
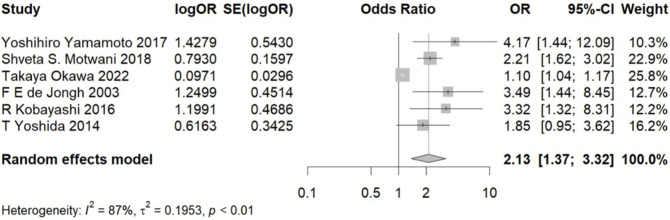
Meta-analysis of the association between hypoalbuminemia and CIA. Note: OR, odds ratio and CI, confidence interval.

Due to high statistical heterogeneity, a random effects model was employed. The forest plot indicates a significant association between hypoalbuminemia and increased risk of CIA (OR, 2.13; 95% CI, 1.37–3.32) (Figure 1). Our sensitivity analysis yielded consistent results, demonstrating that heterogeneity did not originate from a single study, thus affirming the robustness of our analytical findings (Supplementary Figure S3).

### 3.2 Real-world data study

In this study, data were collated from two prominent tertiary healthcare institutions in China. The analysis included 5,452 patients from the Third Xiangya Hospital who were treated with cisplatin (average age 54.1 years; 49.3% female). Among these patients, 143 cases (2.6%) developed CIA. Similarly, at the Hunan Cancer Hospital, 25,214 cisplatin-treated patients (average age 56.2 years; 30.0% female) were studied, with 181 instances (0.7%) of CIA observed. Participant flow diagrams and baseline characteristics for the Third Xiangya Hospital and Hunan Cancer Hospital are detailed in Supplementary Figure S4 and Table 2 respectively.

**TABLE 2:** Baseline characteristics of patients at the Third Xiangya Hospital and Hunan Cancer Hospital.

Characteristic		The Third Xiangya Hospital		*P*	Hunan Cancer Hospital		*P*
		Hypoalbuminemia	Non-hypoalbuminemia		Hypoalbuminemia	Non-hypoalbuminemia	
Age (years), mean (SD)		56.3 (11.8)	53.4 (10.9)	<0.001	56.2 (10.1)	51.7 (10.5)	<0.001
BMI (Kg/m^2^), mean (SD)		21.8 (3.3)	22.8 (3.2)	0.69	21.8 (3.3)	23.2 (3.1)	<0.001
Sex, %	Female	0.5	0.5	0.13	0.3	0.3	0.33
	Male	0.5	0.5		0.7	0.7	
Urea (mmol/L), mean (SD)		4.6 (2.2)	4.8 (1.6)	0.49	4.6 (1.9)	4.9 (1.5)	<0.001
Uric acid (μmol/L), mean (SD)		253.5 (104.9)	310.6 (92.2)	0.58	287.3 (97.9)	336.9 (88.1)	<0.001
Baseline creatinine (μmol/L), mean (SD)		66.5 (25.7)	67.5 (17.6)	0.646	67.2 (20.3)	69.6 (16.0)	<0.001

**FIGURE 2:**
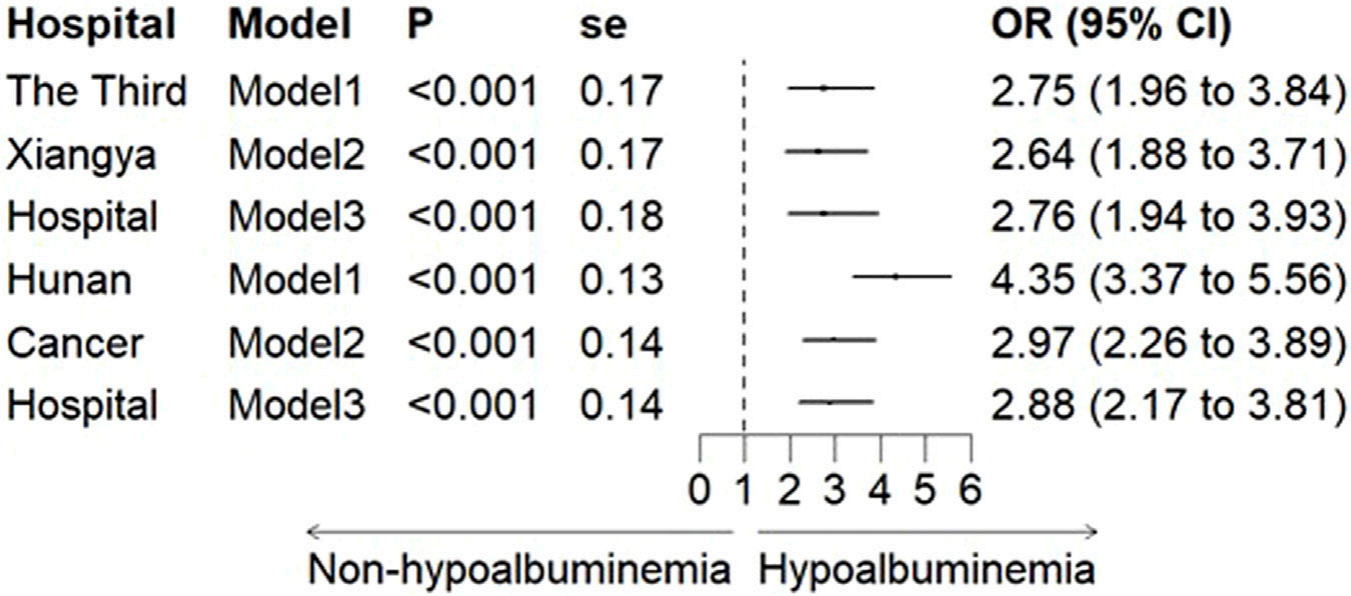
The Association between hypoalbuminemia and CIA in the Third Xiangya Hospital and Hunan Cancer Hospital. Note: Model 1 was unadjusted; Model 2 was adjusted for age, BMI, and gender; Model 3 further adjusted for pre-treatment creatinine, uric acid, and urea. OR: odds ratio, and CI: confidence interval.

**FIGURE 3:**
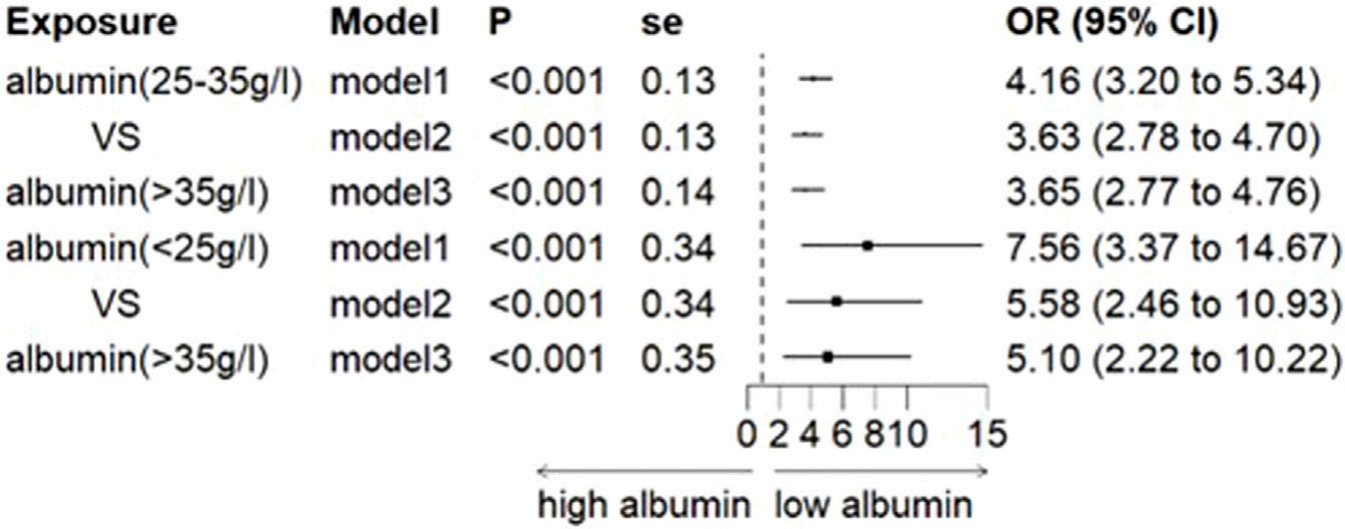
Association between stratified albumin levels and CIA. Note: Model 1 was unadjusted; Model 2 was adjusted for age, BMI, and gender; Model 3 further adjusted for pre-treatment creatinine, uric acid, and urea. OR: odds ratio, and CI: confidence interval.

In both the Third Xiangya Hospital and Hunan Cancer Hospital cohorts, logistic regression models showed a significant association between hypoalbuminemia and increased risk of CIA in all models. After adjusting for all variables, the OR values were 2.76 (95% CI, 1.94–3.93) and 2.88 (95% CI, 2.17–3.81), respectively (Figure 2). Furthermore, when hypoalbuminemia was categorized into mild and severe levels, the results also showed that lower albumin levels correlated with a higher risk of CIA (OR, 3.65; 95% CI, 2.77–4.76 for mild vs. OR, 5.10; 95% CI, 2.22–10.22 for severe) (Figure 3; Supplementary Table S3).

To assess whether the association between hypoalbuminemia and CIA is influenced by specific baseline characteristics, we conducted subgroup analyses and interaction tests, stratifying by age, gender, hypertension, and diabetes. The interaction test results showed that none of the covariates had a significant impact on the association between hypoalbuminemia and CIA (*P* > 0.05) (Table 3). This indicates that the association between hypoalbuminemia and CIA is consistent across all four subgroups, demonstrating the stability of the results.

**TABLE 3:** Association of albumin with CIA.

	OR (95%CI)	*P*	p for interaction
Sex			0.2
male	3.82 (2.74–5.19)	<0.001	
female	2.96 (2.24–3.86)	<0.001	
Age			0.7
<60	3.52 (2.67–4.56)	<0.001	
≥60	3.28 (2.31–4.57)	<0.001	
Hypertension			0.2
1	2.60 (1.42–4.38)	<0.001	
0	3.74 (2.97–4.65)	<0.001	
Diabetes			0.74
1	3.04 (1.05–7.10)	0.02	
0	3.58 (2.88–4.41)	<0.001	

1: Yes, 0: No, for example, for hypertension, 1 represents hypertensive patients, and 0 represents non-hypertensive patients. OR, odds ratio and CI, confidence interval.

## 4 Discussion

This study represents the most comprehensive assessment to date of the association between hypoalbuminemia and CIA, including meta-analysis and dual-center real-world data analysis. Firstly, this study conducted the first meta-analysis on the relationship between hypoalbuminemia and CIA revealing a significant increase in the risk of CIA associated with hypoalbuminemia. And then an analysis of the largest sample from real-world data across two centers further substantiated this association. In summary, this study uniquely confirms that hypoalbuminemia elevates the risk of developing CIA. These findings facilitate risk stratification and personalized management of patients with CIA, potentially reducing the incidence of CIA.

Cisplatin’s nephrotoxicity has always been a major factor limiting its clinical application. Early clinical trials showed a nephrotoxicity incidence rate as high as 50%. Although hydration has significantly reduced cisplatin’s nephrotoxicity, the incidence rate remains high (Mandic et al., 2003; Rybak et al., 2009; Lee et al., 2007; dos Santos et al., 2012; Hoek et al., 2016). Therefore, further exploration of risk factors is needed. Based on the mechanism by which hydration reduces cisplatin’s nephrotoxicity, hydration can reduce the concentration of free cisplatin, thereby reducing CIA. According to a pharmacokinetic study, 65%–98% of intravenously administered cisplatin binds to plasma proteins, especially albumin, within 1 day (Gullo et al., 1980). Therefore, we hypothesize that hypoalbuminemia may be an independent risk factor for CIA, as low serum albumin levels may increase the concentration of free cisplatin, thereby increasing the risk of CIA. Some previous studies have explored the correlation between hypoalbuminemia and CIA, but they did not formally propose hypoalbuminemia as an independent risk factor for CIA, and the existing study results are inconsistent. Yoshida et al. (2014) found that the association between hypoalbuminemia and increased risk of CIA was not statistically significant in a cohort with 496 patients.

We conducted a comprehensive meta-analysis encompassing six studies with a collective total of 4,359 participants. The result demonstrates a significant association between hypoalbuminemia and an increased risk of CIA (OR, 2.13; 95%CI, 1.37–3.32). These studies were observational and, upon evaluation using NOS, were deemed to possess moderate quality evidence. The risk of bias, as assessed by the AHRQ criteria, was also considered moderate. The retrospective design prevalent among these studies, coupled with the presence of small-study effects and significant statistical heterogeneity, could potentially skew results. However, sensitivity analysis through sequential exclusion of individual studies did not alter the overall outcome, affirming the robustness of our findings and enhancing the credibility of our conclusions. Despite the inherent limitations associated with observational research, including potential heterogeneity and bias, our study offers valuable insights into the relationship between hypoalbuminemia and CIA.

From the studies included in the meta-analysis, most of the existing research is single-center with relatively small sample sizes. They have not thoroughly assessed the relationship between different severities of hypoalbuminemia and CIA, nor have they explored the relationship between hypoalbuminemia and CIA in different patient populations. Therefore, we embarked on an analysis of patients treated with cisplatin across two major tertiary hospitals in China—one a comprehensive tertiary hospital and the other a specialized cancer hospital. Incorporating a total of 30,666 cancer patients, this research stands as the most extensive clinical investigation to date into the risk association between hypoalbuminemia and CIA, marking it as the first study of its kind in China.

To reduce the influence of extraneous variables on the outcomes, an initial adjustment was made for covariates including age, gender, and BMI through multivariate analysis. Subsequently, adjustments were extended to renal function indicators such as uric acid, urea, and baseline creatinine levels, acknowledging the consensus in existing literature that baseline renal function acts as an independent risk factor for drug-induced acute kidney injury (DI-AKI). The findings, even after these adjustments, consistently identified hypoalbuminemia as an independent risk factor for CIA across both hospital settings, affirming the reliability of the results. Our study highlights the imperative for implementing additional preventative measures to substantially reduce the risk of CIA occurrence. Additionally, we further explored the association between the severity of hypoalbuminemia and CIA, finding that the more severe the hypoalbuminemia, the higher the risk of CIA. We also conducted subgroup analyses, discovering that the relationship between hypoalbuminemia and CIA was not influenced by age, gender, hypertension, or diabetes. The results remained stable across these subgroups.

Unlike other risk factors, hypoalbuminemia is a risk factor that can be quickly corrected in the short term through the administration of human serum albumin. The risk factors for CIA include age, gender, hypoalbuminemia, dosage of cisplatin, and a history of hypertension. Among these, age and gender are inherent characteristics of patients and thus non-modifiable. The dosage of cisplatin is meticulously calculated based on the patient’s disease requirements; reducing the dosage may compromise therapeutic efficacy, making it a challenging factor to intervene. Hypertension, being a chronic condition, cannot be immediately corrected. Hypoalbuminemia, however, stands out as a condition that can be corrected within a short timeframe. Correcting hypoalbuminemia to prevent other diseases has already shown successful cases. Macedo et al. (2021) evaluated the preventive effect of albumin infusion in reducing the risk of hypotension during dialysis in hospitalized patients with hypoalbuminemia. The results showed that albumin infusion significantly reduced the incidence of hypotension, indicating that hypoalbuminemia is a modifiable risk factor that can be quickly corrected with albumin supplementation. This finding has important implications for the prevention and management of CIA.

Our study possesses several strengths. Given the ethical constraints of prospective research, we conducted the most comprehensive assessment of the association between hypoalbuminemia and CIA. We are the first to undertake a real-world data study with the largest sample size in China, exploring the link between hypoalbuminemia and CIA risk across two distinct types of large tertiary hospitals, thereby representing diverse populations and enhancing the generalizability of our findings. Furthermore, we conducted the initial meta-analysis on hypoalbuminemia and CIA risk. Our study provides a basis for risk stratification among CIA patients, and given that hypoalbuminemia is a modifiable risk factor, it offers recommendations for personalized management and preventive measures, which are crucial for reducing the incidence of CIA.

It is important to acknowledge the limitations of our study. Our research is an observational study based on multiple sources, which may entail reverse causality. However, given the constraints of prospective studies, we conducted a comprehensive assessment using multi-source data, and the consistency of results greatly reduce the possibility of reverse causality. Secondly, there exists considerable statistical heterogeneity among the studies included in our meta-analysis, even though we utilized random-effects models to summarize effect estimates. This point should be duly noted.

## 5 Conclusion

In conclusion, we have confirmed that hypoalbuminemia constitutes an independent risk factor for CIA. Consequently, we recommend proactive adjustment of albumin levels for patients with hypoalbuminemia to reduce the risk of CIA occurrence.

## Data Availability

The original contributions presented in the study are included in the article/Supplementary Material, further inquiries can be directed to the corresponding author.
