# Supplementary material for: Hypoalbuminemia and cisplatin-induced acute kidney injury
Source: Front Pharmacol. 2024 Dec 11;15:1510477. doi: 10.3389/fphar.2024.1510477 (PMC11668559; doi:10.3389/fphar.2024.1510477)
Supplement: Supplementary file 1 [file DataSheet1.docx]

Supplementary Material

# Supplementary Tables

**Table S1. Literature search strategy**

| **Database** | **Search terms** |
| --- | --- |
| **Pubmed** | #1 (Cisplatin OR cis-Diamminedichloroplatinum(II) OR Platinum Diamminodichloride OR Diamminodichloride, Platinum OR cis-Platinum OR cis Platinum OR Dichlorodiammineplatinum OR cis-Diamminedichloroplatinum OR cis Diamminedichloroplatinum OR cis-Dichlorodiammineplatinum(II) OR NSC-119875 OR Platino OR Platinol OR Biocisplatinum OR Platidiam)  #2 (acute kidney injury OR Acute Kidney Injuries OR Kidney Injuries, Acute OR Kidney Injury, Acute OR Acute Renal Injury OR Acute Renal Injuries OR Renal Injuries, Acute OR Renal Injury, Acute OR Renal Insufficiency, Acute OR Acute Renal Insufficiencies OR Renal Insufficiencies, Acute OR Acute Renal Insufficiency OR Kidney Insufficiency, Acute OR Acute Kidney Insufficiencies OR Kidney Insufficiencies, Acute OR Acute Kidney Insufficiency OR Kidney Failure, Acute OR Acute Kidney Failures OR Kidney Failures, Acute OR Acute Renal Failure OR Acute Renal Failures OR Renal Failures, Acute OR Renal Failure, Acute OR Acute Kidney Failure OR Nephrotoxicity)  #3 (Serum albumin OR Albumin, Serum OR Plasma Albumin OR Albumin, Human Serum OR Human Serum Albumin OR Albumin Human OR Human, Albumin OR Human Albumin OR Albumin, Human OR Hypoalbuminemia OR Hypoproteinemia)  #1 AND #2 AND #3 138 results  Period of inclusion: to January 10, 2024 |
| **Embase** | #1 ('cisplatin'/exp OR cisplatin)  #2 ('acute kidney injury'/exp OR 'acute kidney injury' OR (acute AND ('kidney'/exp OR kidney) AND ('injury'/exp OR injury)) OR 'nephrotoxicity'/exp OR nephrotoxicity)  #3 ('serum albumin'/exp OR 'serum albumin' OR (('serum'/exp OR serum) AND ('albumin'/exp OR albumin)) OR 'plasma albumin'/exp OR 'plasma albumin' OR (('plasma'/exp OR plasma) AND ('albumin'/exp OR albumin)) OR 'hypoalbuminemia'/exp OR hypoalbuminemia OR 'hypoproteinemia'/exp OR hypoproteinemia)  #1 AND#2 AND #3 364 results  Period of inclusion: to January 10, 2024 |
| **Web of**  **science** | #1 (Cisplatin OR cis-Diamminedichloroplatinum(II) OR Platinum Diamminodichloride OR Diamminodichloride, Platinum OR cis-Platinum OR cis Platinum OR Dichlorodiammineplatinum OR cis-Diamminedichloroplatinum OR cis Diamminedichloroplatinum OR cis-Dichlorodiammineplatinum(II) OR NSC-119875 OR Platino OR Platinol OR Biocisplatinum OR Platidiam)  #2 (Serum Albumin OR Albumin, Serum OR Plasma Albumin OR Hypoalbuminemia OR Hypoproteinemia)  #3 (acute kidney injury OR Acute Kidney Injuries OR Kidney Injuries, Acute OR Kidney Injury, Acute OR Acute Renal Injury OR Acute Renal Injuries OR Renal Injuries, Acute OR Renal Injury, Acute OR Renal Insufficiency, Acute OR Acute Renal Insufficiencies OR Renal Insufficiencies, Acute OR Acute Renal Insufficiency OR Kidney Insufficiency, Acute OR Acute Kidney Insufficiencies OR Kidney Insufficiencies, Acute OR Acute Kidney Insufficiency OR Kidney Failure, Acute OR Acute Kidney Failures OR Kidney Failures, Acute OR Acute Renal Failure OR Acute Renal Failures OR Renal Failures, Acute OR Renal Failure, Acute OR Acute Kidney Failure OR Nephrotoxicity)  #1 AND#2 AND #3 210 results  Period of inclusion: to January 10, 2024 |

**Table S2 Grading of evidence included in meta-analysis**

| Study | Selection | Comparability | Exposure (Outcome) | Quality score |
| --- | --- | --- | --- | --- |
| Yoshihiro Yamamoto 2017^[13]^ | ** | * | ** | 5 |
| Shveta S. Motwani 2018^[14]^ | ** | ** | ** | 6 |
| Takaya Okawa 2022^[15]^ | ** | * | ** | 5 |
| F E de Jongh 2003^[16]^ | ** | * | ** | 5 |
| R Kobayashi 2016^[20]^ | ** | * | ** | 5 |
| T Yoshida 2014^[18]^ | ** | * | ** | 5 |

**Table S3 Logistic regression analysis of patients from the Third Xiangya Hospital and Hunan Cancer Hospital**

| Characteristic | Model | OR | CI | P |
| --- | --- | --- | --- | --- |
| Sex | Model2 | 0.515 | 0.411-0.645 | * |
|  | Model3 | 0.978 | 0.750-1.276 | 0.873 |
| Age | Model2 | 0.986 | 0.982-0.991 | * |
|  | Model3 | 0.985 | 0.981-0.990 | * |
| BMI | Model2 | 0.980 | 0.947-1.015 | 0.263 |
|  | Model3 | 0.996 | 0.961-1.031 | 0.812 |
| Baseline creatinine | Model3 | 0.950 | 0.941-0.960 | * |
| Urea | Model3 | 1.218 | 1.139-1.300 | * |
| Uric acid | Model3 | 1.001 | 0.999-1.002 | 0.321 |

*P<0.05

# Supplementary Figures


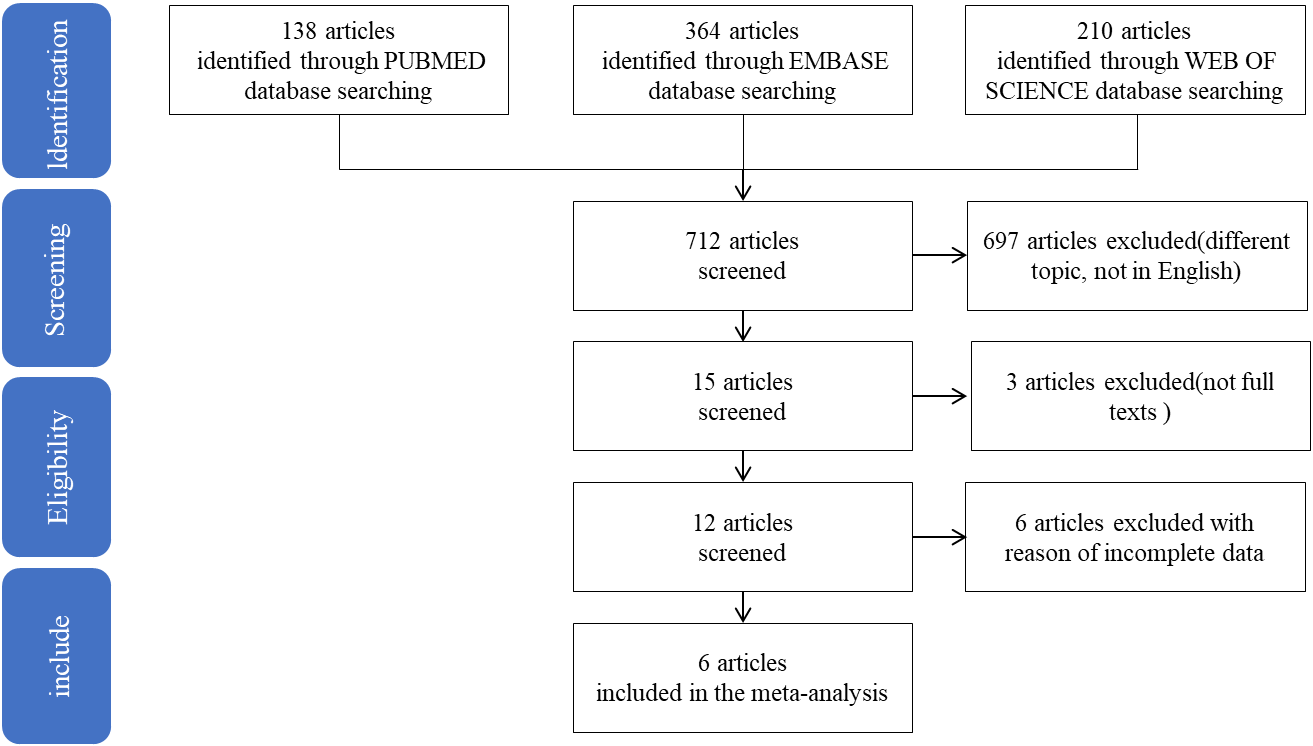


**Figure S1 Flow chart of included literature.**


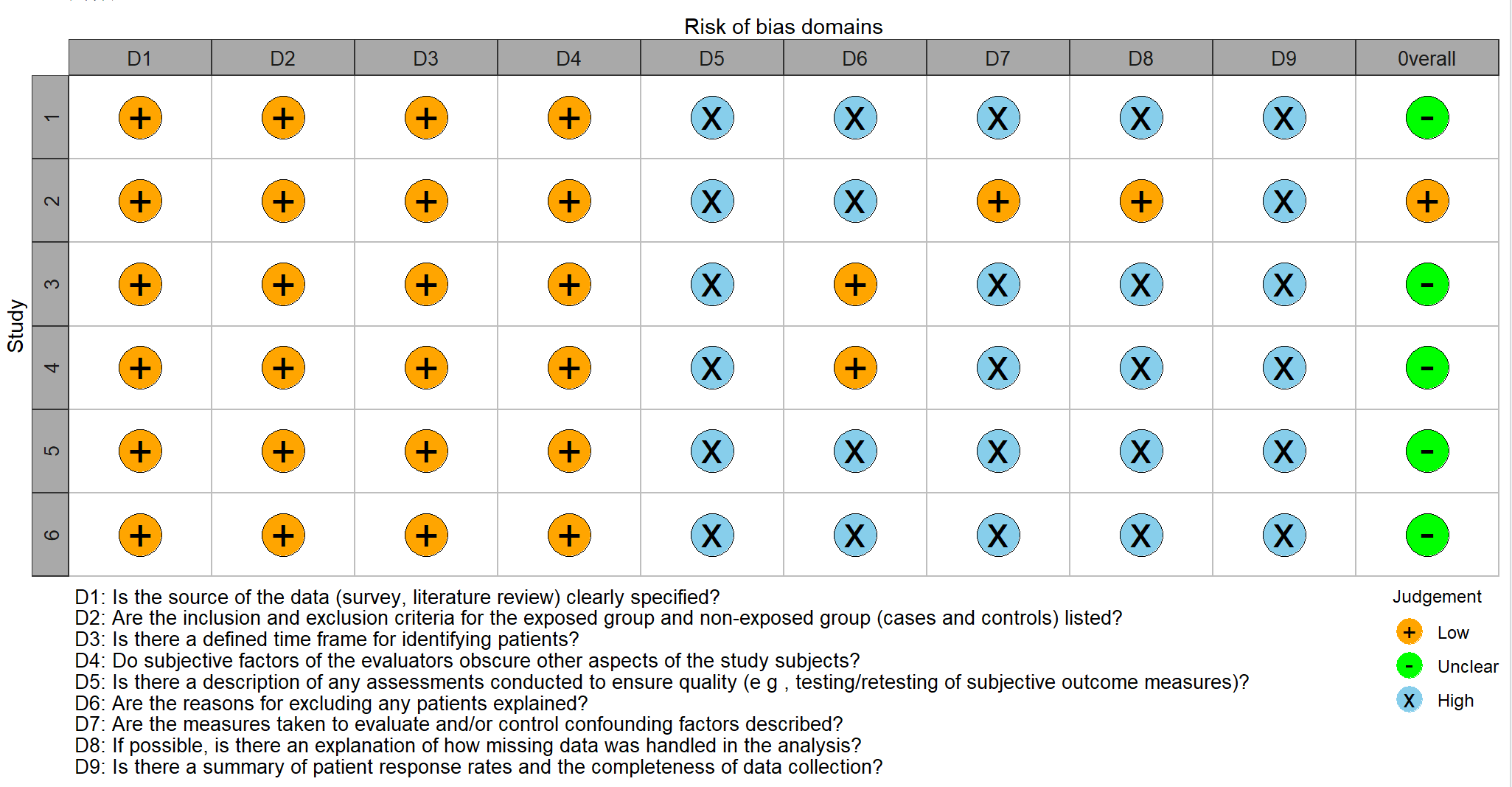


**Figure S2 Summary of risk of bias using AHRQ**


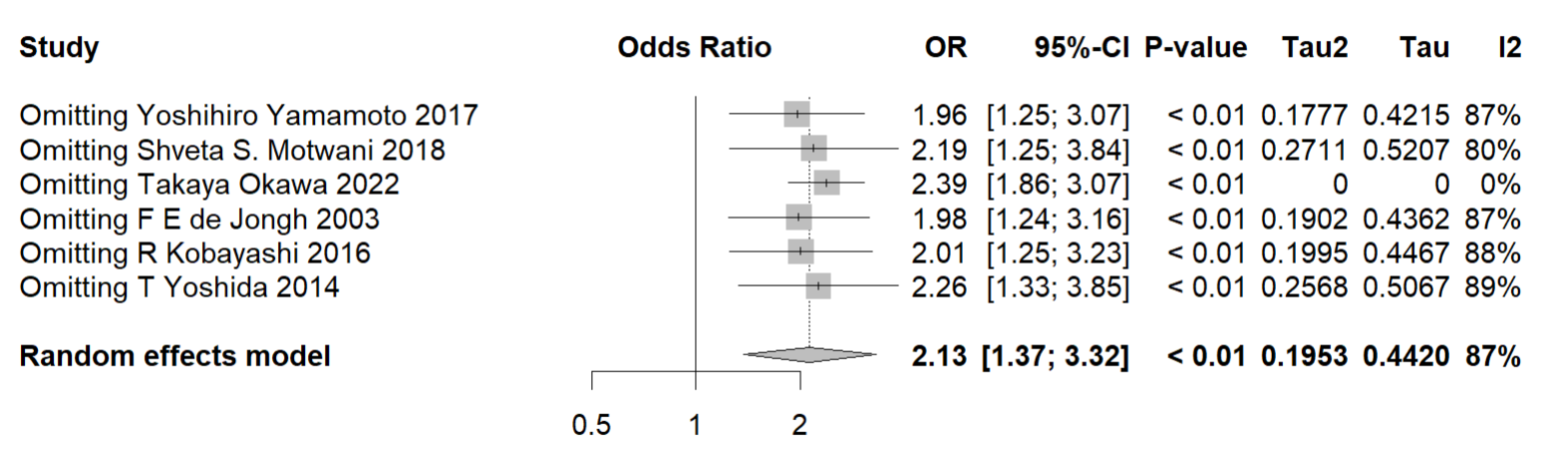


**Figure S3 Results of leave-one-out analysis: meta-analysis**


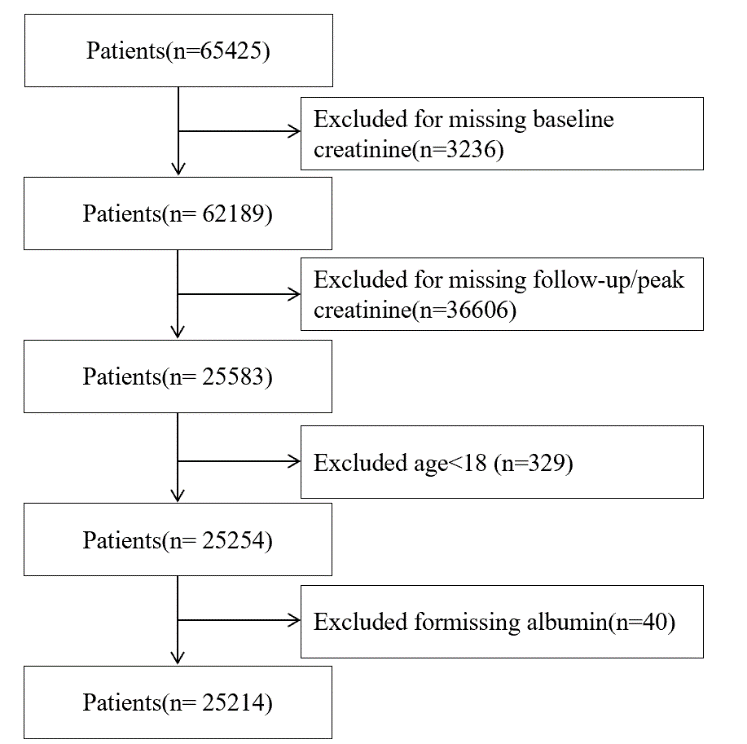


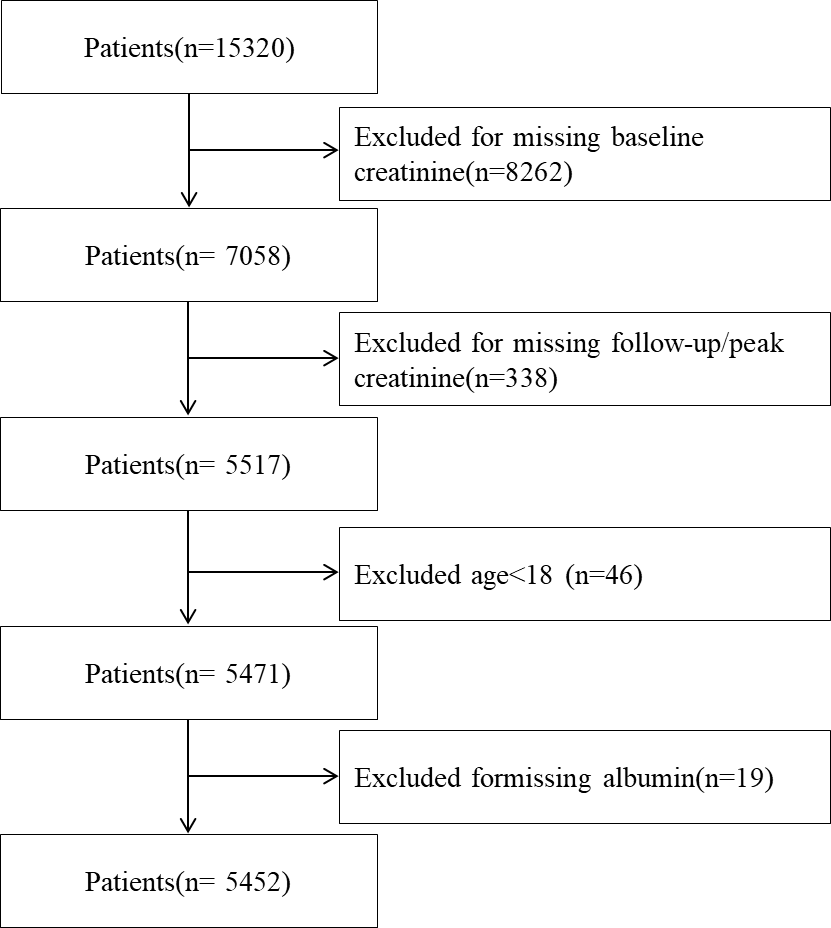


**Figure S4 Participant flowchart for the Third Xiangya Hospital and Hunan Cancer Hospital**
